# Comparison of a new biometer using swept-source optical coherence tomography and a conventional biometer using partial coherence interferometry

**DOI:** 10.1371/journal.pone.0196401

**Published:** 2018-04-24

**Authors:** Tomoaki Higashiyama, Hazuki Mori, Fumi Nakajima, Masahito Ohji

**Affiliations:** Department of Ophthalmology, Shiga University of Medical Science, Otsu, Shiga, Japan; National Yang-Ming University Hospital, TAIWAN

## Abstract

The aim of this study was to compare the axial lengths (ALs) using a new biometer with swept-source optical coherence tomography (Argos) versus ALs using a conventional biometer with partial coherence interferometry (IOL Master, version 5). The ALs in 48 eyes of 48 cataract patients were measured with Argos using refractive indexes that correspond to the particular tissue and with IOL Master using a single refractive index. The eyes were divided into three subgroups by AL length: short-AL group (n = 16), <23.27 mm; intermediate-AL group (n = 16), 23.27–24.03 mm; long-AL group (n = 16), ≥24.04 mm. The ALs (mm) measured with the Argos and IOL Master biometers, respectively, were 22.77 ± 0.43 and 22.74 ± 0.44, 23.63 ± 0.21 and 23.62 ± 0.21, and 26.00 ± 1.61 and 26.05 ± 1.64 in the short-, intermediate-, and long-AL groups, respectively. The mean ALs with the Argos biometer were longer than those with the IOL Master biometer in the short-AL group (*P* = 0.002) There was no significant difference in the intermediate-AL groups (*P* = 0.14). In contrast, the mean ALs with the Argos biometer were shorter than those with the IOL Master biometer in the long-AL group (*P* < 0.001). Differences between the ALs measured with the two biometers were statistically significant in short- and long-AL subgroups. However, the differences might not be clinically significant.

## Introduction

The measurement of axial length (AL) is one of the most important examinations for cataract surgery. To choose the appropriate intraocular lens (IOL) power, the AL measurement must be accurate. The AL has been measured using optical biometry and/or ultrasonography [1–7]. Optical biometry is the more accurate method for these measurements [8]. IOL Master (version 5; Carl Zeiss, Jena, Germany), an optical biometer that uses partial coherence interferometry (PCI) with the light source centered at 780 nm, is widely used. The AL, from the corneal surface to the retinal pigment epithelium, with this biometer is measured using only a single refractive index, although the refractive index of each tissue is different.

The Argos (Suntec, Inc., Aichi, Japan) is a new biometer that uses swept-source optical coherence tomography (SS-OCT) with the light source centered at 1050 nm [9, 10]. The use of SS-OCT improves the signal-to-noise ratio because the narrow-bandwidth wavelength light source improves tissue penetration and hence image quality [11, 12]. Thus, a biometer using SS-OCT could better measure the AL in eyes with a severe cataract than conventional biometers [13–16]. With Argos, ALs are measured from the corneal surface to the retinal pigment epithelium using refractive indexes that correspond to each tissue, so the AL is the sum of four lengths in the four segments. Thus, Argos is different from the IOL Master (version 5) in two ways: It uses SS-OCT, and its measurements are performed using refractive indexes that correspond to each tissue.

Shammas et al. also compared the Argos and IOL Master biometers and found a mean difference in the ALs of only 0.01 mm between the two biometers [9]. However, their comparison did not address whether the ALs were short or long. The purpose of the present study was to make these comparisons of the two biometers according to short or long ALs.

## Methods

We conducted a retrospective review of the medical records of 55 eyes in 55 patients (31 women, 24 men; mean age 72.9 ± 8.2 years) who underwent cataract surgery at the Department of Ophthalmology, Shiga University of Medical Science Hospital from April 2017 to May 2017. Their mean logMAR best corrected visual acuity was 0.52 ± 0.51. Eyes with a history of (1) ocular/refractive surgery or (2) corneal or retinal disease were excluded from the study.

The institutional review board of Shiga University of Medical Science approved this retrospective study. The study adhered to the tenets of the Declaration of Helsinki. The ethics committee stated that the patients' informed consent was not needed because the examinations were routinely performed and the data extracted from the patients’ medical records.

The ALs in all eyes were measured using the two biometers—Argos and IOL Master (version 5)—on the same day. Only the first operated eye was used in each patient to avoid data duplication as was seen in another comparative study [9].

The Argos, with SS-OCT, has an A-scan rate of 3000 lines/sec, and the light source is centered at 1050 nm. Patients were instructed to fixate on the biometer's internal fixation target. The lengths of four segments (corneal thickness, aqueous depth, lens thickness, thickness of the vitreous humor to the retina) were measured with Argos using a refractive index corresponding to each tissue (cornea, 1.374; aqueous humor, 1.336; lens, 1.410; vitreous humor, 1.336). The AL was calculated as the sum of the distances of the four segments. Measurements with Argos were considered successful when the lens and retina were shown clearly in a captured two-dimensional image of the whole eye. Because the value of vitreous cavity length was not revealed in the Argos data, the values in this article were calculated by subtracting the anterior chamber depth and lens thickness from the AL.

The IOL Master (version 5), with PCI, has the wavelength of 780 nm. Patients were instructed to fixate on the biometer's internal fixation target. The AL from the cornea to the retina was measured with the IOL Master using a single refractive index. IOL Master measurements with a signal-to-noise ratio >2 were considered successful, similar to that of a protocol reported in previous studies [17, 18].

In the comparative analysis of ALs, the eyes were divided into three subgroups according to the AL (i.e., short, intermediate, long). The number and range of mean ALs of two biometers measurements in each group were as follows: short-AL group (n = 16), <23.27 mm; intermediate-AL group (n = 16), 23.27–24.03 mm, (3) long-AL group (n = 16), ≥24.04 mm. The patients’ characteristics in each subgroup are shown in Table 1.

**Table 1:** Patients’ characteristics, by AL subgroups.

	Number (eyes)	AL in Argos (range, mm)	AL in IOM Master (range, mm)
Short AL	16	21.72–23.29	21.68–23.24
Intermediate AL	16	23.22–24.03	23.33–24.01
Long AL	16	24.04–29.68	24.05–29.82

AL = axial length

### Statistical analysis

All statistical analyses were performed using SPSS Statistics 22 software (IBM, Armonk, NY, USA). The normality of the numerical variables was evaluated using the Shapiro–Wilk test. A paired *t*-test or the Wilcoxon signed-rank test was used to compare the ALs acquired with the two biometers. Pearson’s product–moment correlation coefficient or Spearman’s rank correlation coefficient was used to analyze the correlation between the ALs acquired with the two biometers. The values are expressed as means ± standard deviation. A value of *P* < 0.05 indicates statistical significance.

## Results

### Comparison of success rate

The success rate for AL measurements was 98.2% (54/55 eyes) with Argos and 87.3% (48/55 eyes) with IOL Master. One eye could not be successfully measured in either biometer because of the maturity of the cataract. ALs of six eyes could not be successfully measured with IOL Master but were successfully measured with Argos. These six eyes had moderate-to-severe nuclear cataracts. ALs of the other 48 eyes were successfully measured with both Argos and IOL Master.

### Comparison of ALs

**Fig 1:**
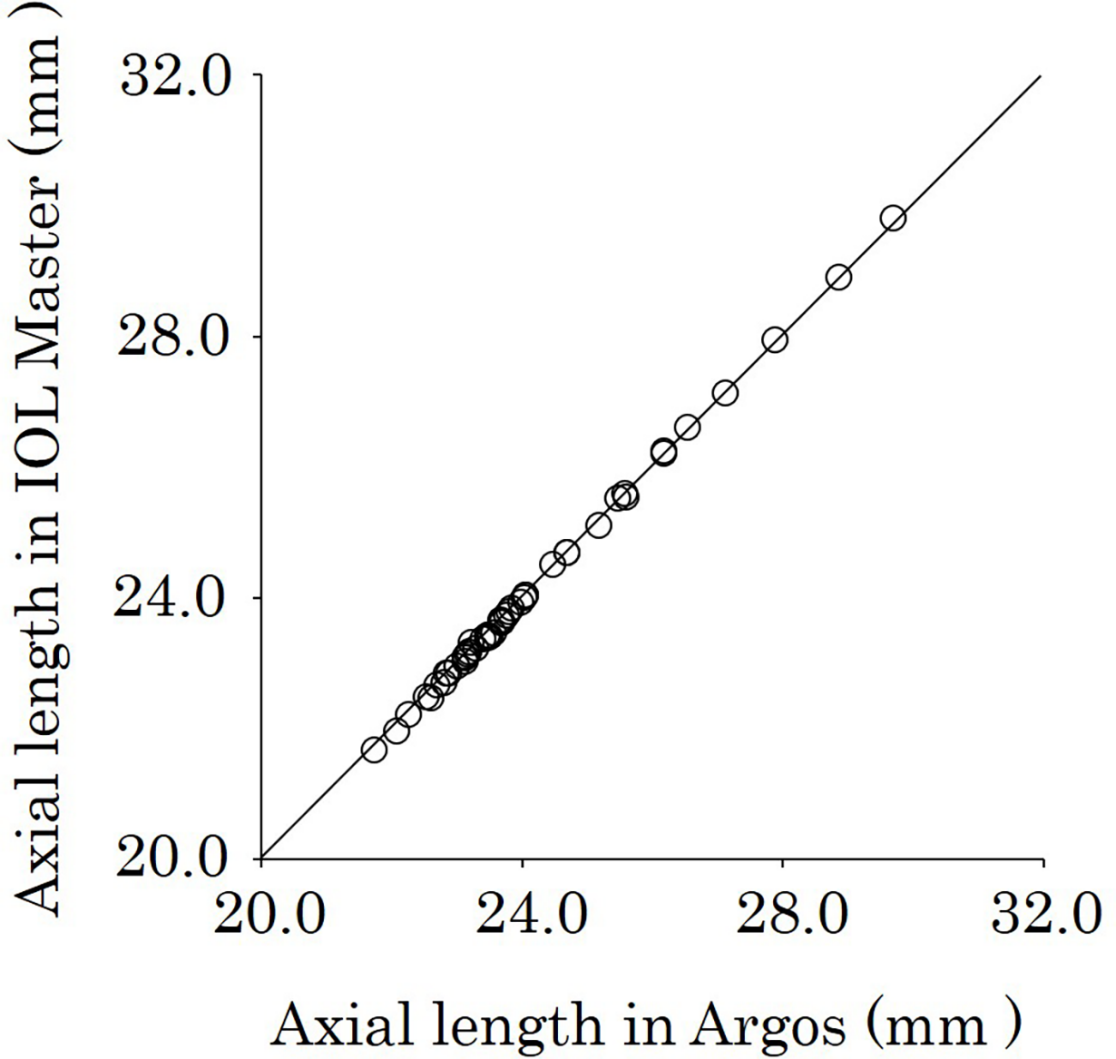
Correlation of AL between Argos and IOL Master biometers. Significant positive correlations were observed between the biometers (*r* = 0.998, *P <* 0.001).AL = axial length.

**Fig 2:**
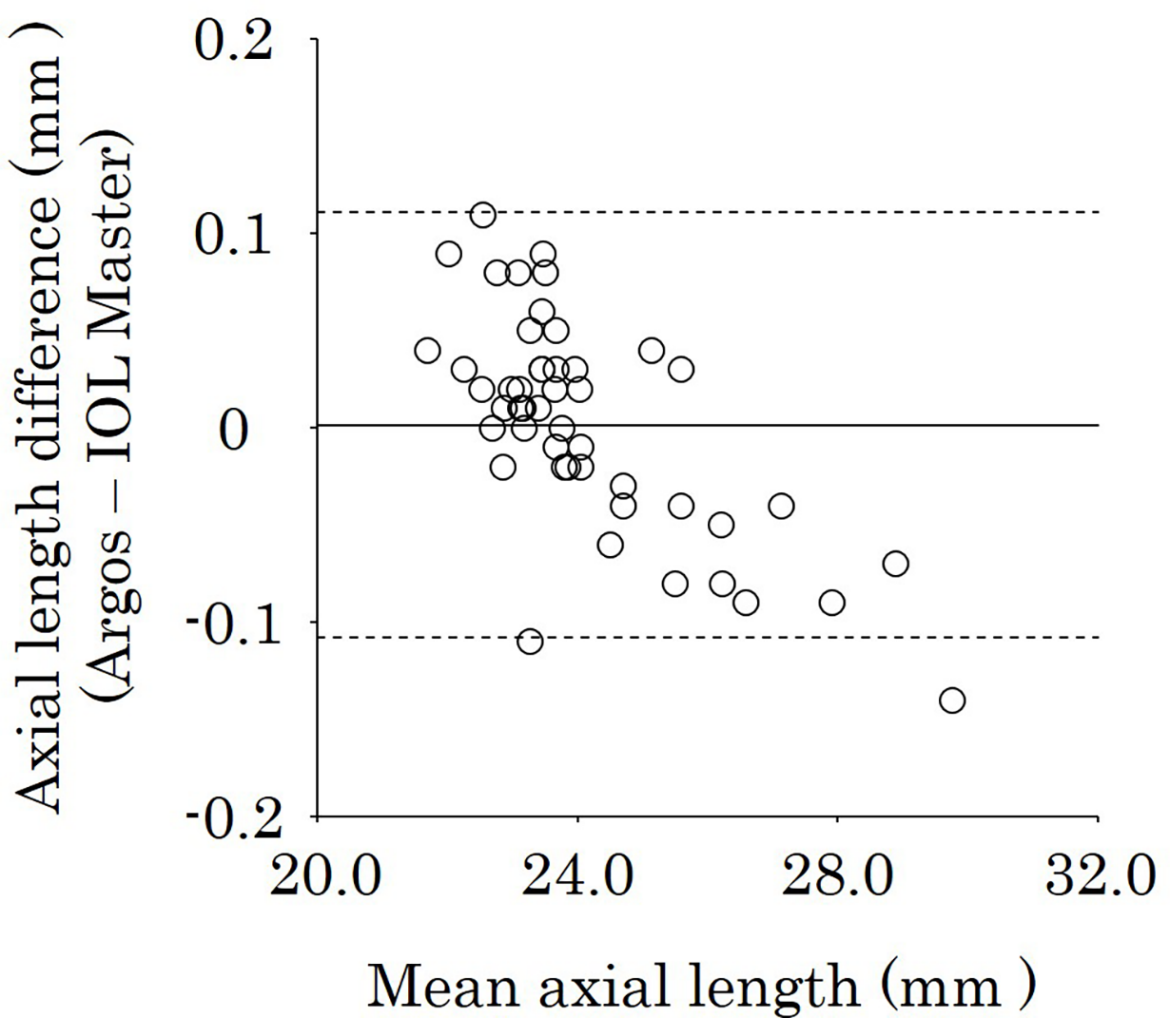
Bland–Altman plot of AL using Argos and IOL Master biometers. The limits of agreement were set at ± 1.96 × standard deviation (SD). There was a significant negative correlation between the mean AL of the two biometers and the AL difference (*r* = -0.63, *P <* 0.001). AL = axial length.

The 48 eyes that could be measured successfully with both biometers were further analyzed. The mean AL was 24.14 ± 1.68 mm with Argos and 24.13 ± 1.71 mm with IOL Master. There was no significant difference between the measurements (*P* = 0.67), and there was a significant positive correlation between the biometers (*r* = 0.998, *P <* 0.001) (Fig 1). Fig 2 shows the Bland–Altaman plot of axial length using Argos and IOL Master. The mean AL difference between two biometers was 0.001 and the limits of agreement were -0.11 to 0.11. There was a significant negative correlation between the mean AL of the two biometers and the AL difference (r = -0.63, *P <* 0.001).

**Fig 3:**
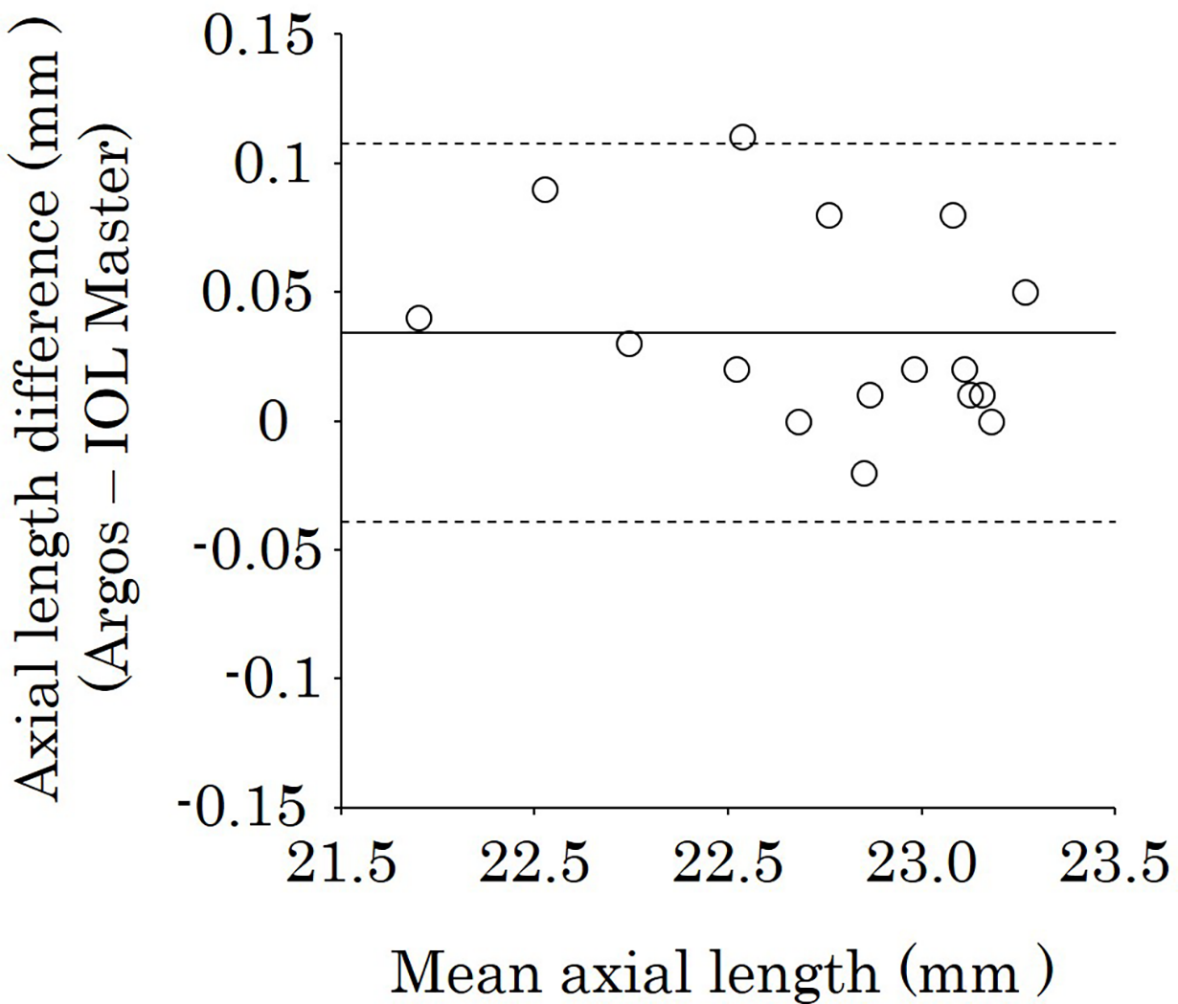
Bland–Altman plot of AL using Argos and IOL Master biometers in the short-AL group. The limits of agreement were set at ± 1.96 × SD. The values with Argos were longer than those with IOL Master in 81.3% (13/16) of eyes in the short-AL group. AL = axial length.

**Fig 4:**
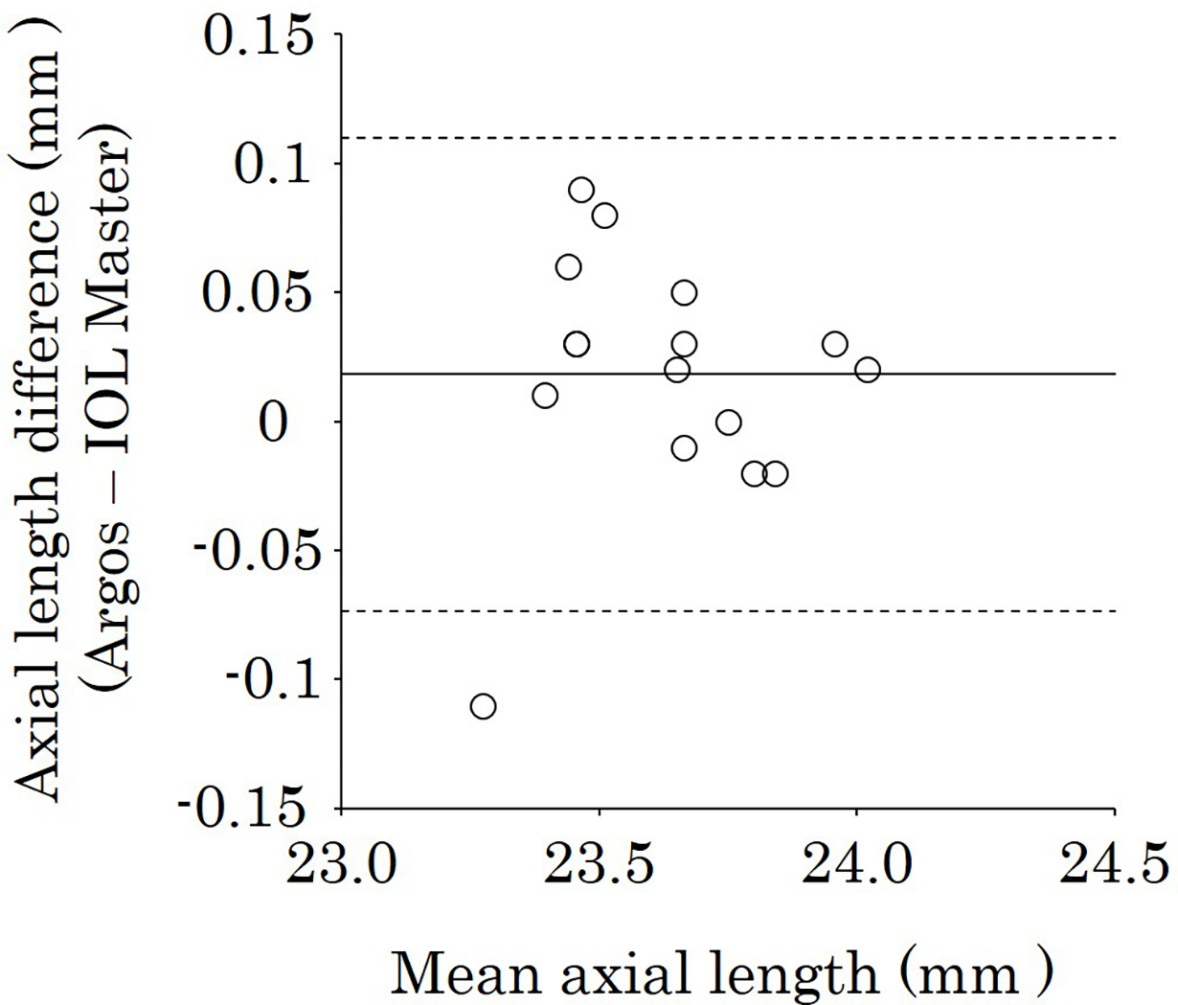
Bland–Altman plot of AL using Argos and IOL Master biometers in the intermediate-AL group. The limits of agreement were set at ± 1.96 × SD. AL = axial length.

**Fig 5:**
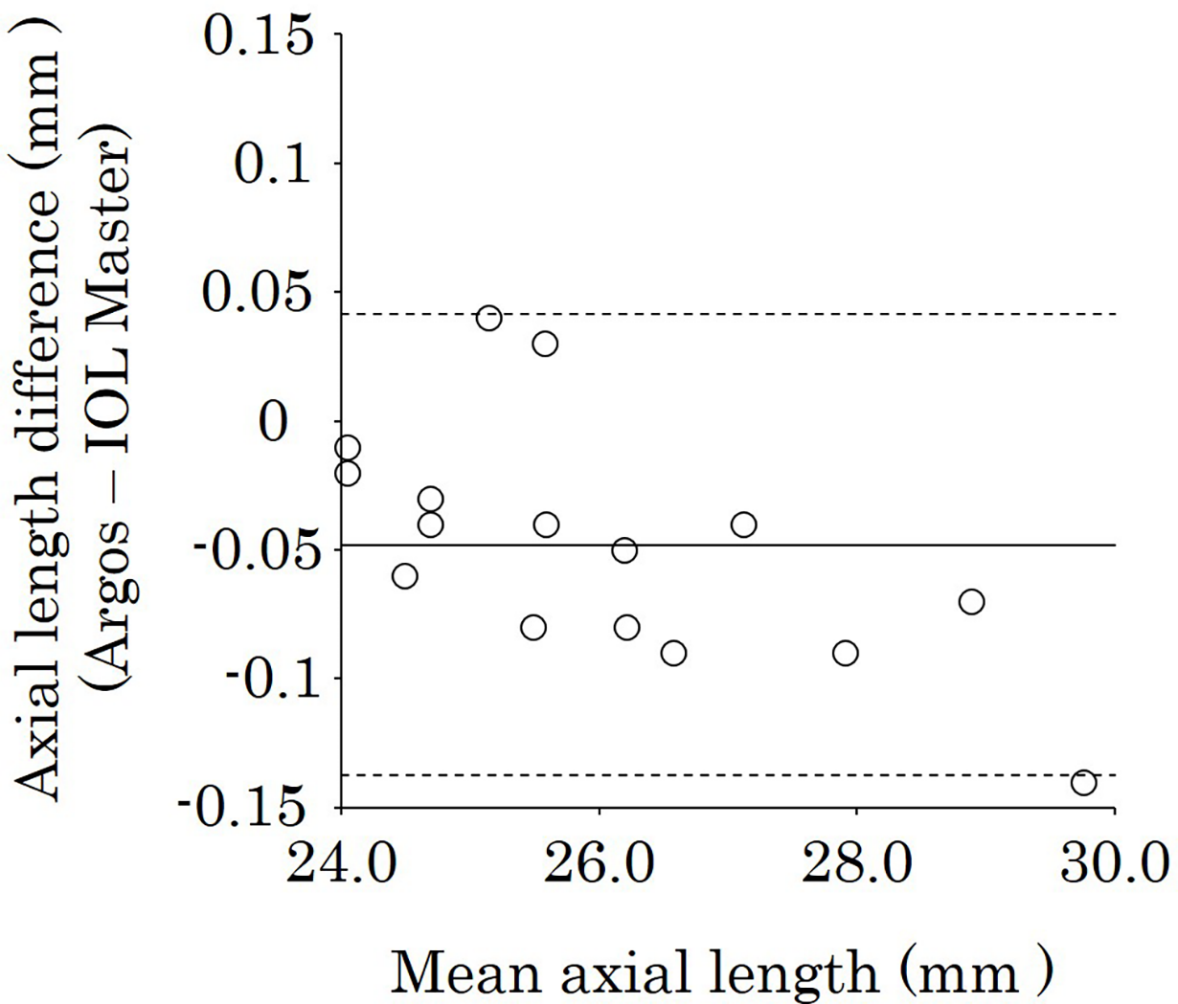
Bland–Altman plot of AL using Argos and IOL Master biometers in the long-AL group. The limits of agreement were set at ± 1.96 × SD. The values with Argos were shorter than those with IOL Master in 87.5% (14/16) of eyes in the long-AL group. AL = axial length.

In a subgroup analysis, the ALs found with Argos and IOL Master were, respectively, 22.77 ± 0.43 mm and 22.74 ± 0.44 mm in the short-AL group, 23.63 ± 0.21 mm and 23.62 ± 0.21 mm in the intermediate-AL group, and 26.00 ± 1.61 mm and 26.05 ± 1.64 mm in the long-AL group (Table 2). The mean values for each tissue in the subgroups by Argos are shown in Table 3. The mean AL differences were slight in all subgroups: 0.03, 0.02, and 0.05 mm in the short-, intermediate-, and long-AL groups, respectively. The mean ALs with Argos were longer than those with IOL Master in the short-AL groups (*P* = 0.002) (Table 2). The ALs measured with Argos were longer than those with IOL Master in 81.3% (13/16) of eyes in the short-AL group (Table 4). There was no significant difference in the intermediate-AL group (*P* = 0.14). In contrast, the mean AL measured with Argos was shorter than that measured with IOL Master in the long-AL group (*P* < 0.001) (Table 2). The ALs with Argos were shorter than those with IOL Master in 87.5% (14/16) of eyes in the long-AL group (Table 4). Figs 3, 4 and 5 show the Bland-Altman plots of axial length using Argos and IOL Master in the short-, intermediate-, and long-AL groups. The limits of agreement were -0.04 to 0.11, -0.07 to 0.11, -0.14 to 0.04 in the short-, intermediate-, and long-AL groups, respectively.

**Table 2:** Axial length differences, by biometer.

	Mean AL in Argos (mm)	Mean AL in IOL Master (mm)	P value	Mean AL difference (Argos- IOL Master, mm)
Short AL	22.77 ± 0.43	22.74 ± 0.44	0.002	0.03
Intermediate AL	23.63 ± 0.21	23.62 ± 0.21	0.14	0.02
Long AL	26.00 ± 1.61	26.05 ± 1.64	< 0.001	- 0.05

AL = axial length

**Table 3:** Mean value of each tissue in subgroups by Argos.

	Mean ACD (mm)	Mean LT (mm)	Mean VCL (mm)
Short AL	2.97 ± 0.32	4.59 ± 0.42	15.21 ± 0.45
Intermediate AL	3.29 ± 0.36	4.48 ± 0.36	15.86 ± 0.34
Long AL	3.53 ± 0.51	4.38 ± 0.36	18.09 ± 1.57

ACD = anterior chamber depth, LT = lens thickness, VCL = vitreous cavity length

**Table 4:** Numbers of eyes evaluated by the two biometers, by AL subgroup.

	Argos > IOL Master	Argos = IOL Master	Argos < IOL Master
Short AL	13 (81.3%)	2 (12.5%)	1 (6.3%)
Intermediate AL	11 (68.8%)	1 (6.3%)	4 (25.0%)
Long AL	2 (12.5%)	0 (0%)	14 (87.5%)

AL = axial length

## Discussion

There was a significant negative correlation between the mean ALs of the two biometers and the AL difference in all patients. The mean ALs measured with Argos were significantly longer than those measured with IOL Master in the short-AL groups. In contrast, in the long-AL group, the mean AL with Argos was significantly shorter than that measured with IOL Master. The previous studies reported the agreement of AL measurements between SS-OCT and PCI [13, 19]. Huang et al reported that the agreement between OA-2000 (SS-OCT biometer; Tomey, Nagoya, Japan) and IOL Master (version.5.4; PCI biometer) was good [19]. Srivannaboon et al reported that the agreement between IOL Master 700 (SS-OCT biometer) and IOL Master 500 (PCI biometer) was also good [13]. However, the previous studies had not analyzed the data according to subgroups based on AL.

The AL measured with Argos was the sum of four lengths in four segments, which were measured using a corresponding refractive index, whereas that with IOL Master was measured using only a single refractive index. The difference in measurement methods might cause the AL difference between the two biometer measurements in this study. A previous study reported that the AL difference between individuals in adults was due mainly to differences in the vitreous cavity [20]. In the present study, there was a significant negative correlation between the mean AL of the two biometers and the AL difference. In addition, the ALs determined with Argos were longer than those with IOL Master in the short-AL groups and shorter in the long-AL group. This AL difference between the two biometers' results might have been caused by differences in the length of the vitreous cavity.

The mean differences in this study were slight in the short-AL groups (0.03 mm) and in the long-AL groups (0.05 mm). A previous study reported that a 0.1 mm error in AL measurement would result in a 0.25 diopter error in IOL power based on the SRK formula [21]. Others reported that a 0.1-mm error in AL measurement is equal to refraction prediction errors of about 0.27 diopter when assuming normal eye dimensions [22, 23]. Applying these reported figures to the current study results, up to 0.135 diopter in the IOL calculation could be caused by the difference reported herein. The AL difference between the two biometers might not be clinically significant because the difference is much less than 0.5 diopter, which is the IOL power step of commercially available IOL.

The success rates for the AL measurements in this study were 98.2% with Argos and 87.3% with IOL Master. In addition, no eyes were successfully measured with IOL Master alone, and IOL Master was unable to measure six eyes successfully. Thus, measuring ALs using a biometer with SS-OCT might be more useful than measuring them with a conventional biometer. A possible reason for the success rate with Argos is its inclusion of SS-OCT, which improved the signal-to-noise ratio compared with that with a conventional biometer [11, 12]. Shammas et al. reported that the success rate of AL measurements was 96% with Argos (SS-OCT biometer) and 77% with IOL Master 500 (PCI biometer) [9]. Srivannaboon et al. also reported that each of 100 eyes could be measured with IOL Master 700 (SS-OCT biometer), whereas five eyes could be not measured with IOL Master 500 (PCI biometer) [13]. Their findings were in agreement with our results.

There are limitations to the present study. First, the number of subjects was small, with only 55 eyes included in the study. Second, this study did not investigate refraction after cataract surgery. Thus, it is necessary to confirm which biometer could provide more reliable cataract surgery in future studies. Third, there could be a bias from dividing the eyes into the three sub-groups. Other previous studies used other ranges of AL such as a short-AL group < 22 mm [24–26]. Because the number of eyes in the short-AL group was small (n = 1) in the range (AL < 22 mm), the number of patients was almost equally divided into three subgroups.

In conclusion, AL measurements with a biometer using SS-OCT would be more useful than with a conventional biometer according to its higher success rate in this study. Based on the AL measurements, there was a statistically significant difference between the two biometers in all subgroups. Even so, the difference might not be clinically significant.

## Supporting information

S1 Table Specific dataset for all individuals. (XLSX)
